# Effectiveness and safety of proton pump inhibitors for treating acute pancreatitis: A protocol for systematic review and meta analysis

**DOI:** 10.1097/MD.0000000000024808

**Published:** 2021-02-26

**Authors:** Tao Cheng, Bo-Fu Liu, Tian-Yong Han, Zhi-Han Gu, Pan Pan, Haifang Yu

**Affiliations:** a Emergency Department; b Laboratory of Emergency Medicine, West China Hospital, Sichuan University; c Disaster Medical Center, Sichuan University, Chengdu, Sichuan, China.

**Keywords:** acute pancreatitis, duration of hospital stays, meta-analysis, mortality, prognosis, proton pump inhibitors, systematic review

## Abstract

**Background:**

Previous studies have showed that anti-acid therapy with proton pump inhibitors (PPIs) can inhibit pancreatic secretion and it may be used in treating acute pancreatitis (AP). But at present, there is no systematic reviews for the evidence and the therapeutic effectiveness and safety of anti-acid therapy with PPIs in AP were not unclear. Therefore, we will undertake a systematic review of the literature to summarize previous evidence regarding this topic, in order to clarify the effectiveness and safety of anti-acid therapy with PPIs in AP.

**Methods:**

We will search the EMBASE, WANFANG DATA, Web of Knowledge, China National Knowledge Infrastructure, PubMed, ClinicalTrials.gov and Cochrane Library from inception to June 30,2021 to retrieve relevant studies using the search strategy: (“Proton pump inhibitors” OR “PPI” OR “PPIs” OR “Omeprazole” OR “Tenatoprazole” OR “Pantoprazole” OR “acid suppression therapy” OR “acid suppression drugs”) AND (“pancreatitis” OR “pancreatitides”). Two authors independently judged study eligibility and extracted data. Heterogeneity will be examined by computing the Q statistic and I^2^ statistic.

**Results:**

This study assessed the efficiency and safety of proton pump inhibitors for treating acute pancreatitis.

**Conclusions:**

This study will provide reliable evidence-based evidence for the clinical application of PPIs for treating AP.

**Ethics and dissemination:**

Ethical approval is unnecessary as this protocol is only for systematic review and does not involve privacy data. The findings of this study will be disseminated electronically through a peer-review publication or presented at a relevant conference.

## 1 Introduction

Acute pancreatitis (AP) is a sudden inflammatory process in the pancreas with variable involvement of nearby organs or other organ systems,^[1–3]^ and the incidence of AP is 34 per 100,000 among human beings, and it is rising worldwide.^[4]^ In the United States, acute pancreatitis leads to 270,000 hospital admissions annually, which is part of one of the leading causes of hospitalization among gastrointestinal diseases^[5]^ and inpatient costs exceed 2.5 billion dollars.^[6]^ Despite improvements in critical care, the mortality of patients with AP remains high, and it is about 2% and 20%.^[7–9]^ Therefore, it is necessary to improve the ability of treating patients with AP.

Previous studies have showed that pantoprazole possesses anti-inflammatory in vivo properties and attenuates the course of AP,^[10]^ and anti-acid therapy with proton pump inhibitors (PPIs) can protect the upper gastrointestinal (GI) mucosa and inhibit pancreatic secretion resting the inflamed pancreas. Nowadays, it is routinely administered in clinical practice in the majority of patients with AP which might be beneficial if it decreases severity or mortality, however, it can be harmful as it might increase the risk for GI infections^[11–14]^ and the current AP guidelines do not include any information regarding the administration of PPIs in AP.^[15–17]^ Therefore, we designed this systematic review and meta-analysis to evaluate the effectiveness and safety of anti-acid therapy with PPIs in AP.

## 2 Methods and analysis

### 2.1 Registration

This protocol of systematic review and meta-analysis is based on the Preferred Reporting Items for Systematic Reviews and meta-analysis Protocols statement guidelines. And the protocol has been registered on International Prospective Register of Systematic Reviews database. The registration number was INPLASY202110048.

### 2.2 Eligibility criteria

The inclusion criteria for the study will include:

(1) studies with patient age ≥18 years old, a minimum hospital stay of 24 h and a diagnosis of AP;
(2) conference abstracts were only included when they provided adequate relevant information for assessment;
(3) the patients with AP was divided into 2 groups (treated with PPI or without PPI);

Exclusion criteria will include: age <18 years old, patients with chronic pancreatitis or pancreas carcinoma and patients with incomplete data.

### 2.3 Searching strategy

**Figure 1:**
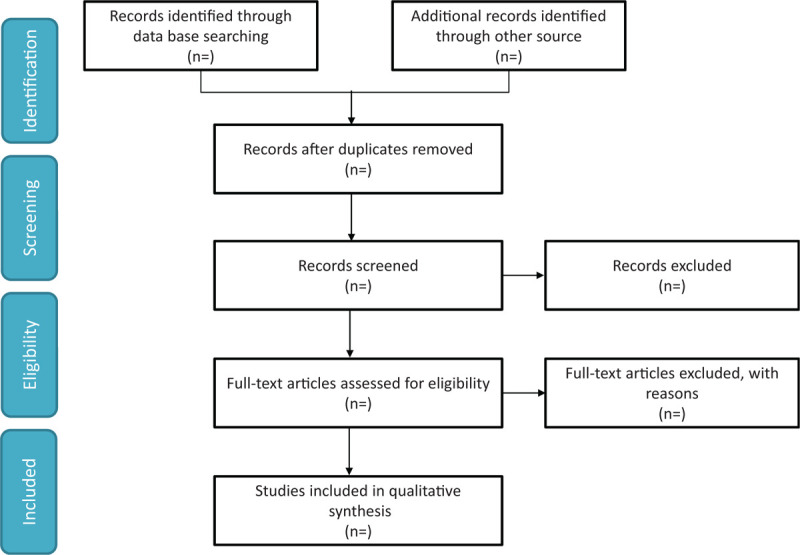
A flow diagram demonstrating the search strategy and study selection process for this study.

We will search the EMBASE, WANFANG DATA, Web of Knowledge, China National Knowledge Infrastructure, PubMed, ClinicalTrials.gov and Cochrane Library from inception to June 30,2021 to retrieve relevant studies using the search strategy: (“Proton pump inhibitors” OR “PPI” OR “PPIs” OR “Omeprazole” OR “Tenatoprazole” OR “Pantoprazole” OR “acid suppression therapy” OR “acid suppression drugs”) AND (“pancreatitis” OR “pancreatitides”). No language restrictions will be applied. We will also search citations of relevant primary and review. Authors of abstract in the meeting will be further searched in PubMed for potential full articles. To minimize the risk of publication bias, we will conduct a comprehensive search that included strategies to find published and unpublished studies. The research summary of the screening flow chart is shown in Figure 1.

### 2.4 Data extraction and Risk of bias

Two reviewers will be employed the searching strategy respectively, by reading the papers and scoring them according to the QUADAS-2 checklist^[18]^ and Newcastle–Ottawa Quality Assessment Scale^[19]^; disagreement will be settled by a third opinion. Important information will be abstracted from the included articles in a standardized form by 2 reviewers. Important information include the name of the first author, publication year, publication country, type of study, study population, sample size, using of PPIs and outcomes studied (hospital mortality and duration of hospital stays). Risk of bias assessment will be carried out according to the Newcastle-Ottawa Scale to rate the internal validity of the individual studies, and funnel plots will be constructed to assess the risk of publication bias.

### 2.5 Statistical analysis

All pairwise meta-analytic calculations will be performed with Review Manager software (RevMan) version 5.3 (Cochrane Collaboration). Heterogeneity will be examined by computing the Q statistic and I^2^ statistic, and presence of reporting bias by visual inspection of funnel plots. Statistical significance was considered when the *P* value <.05.

## 3 Discussion

Acute pancreatitis is a sudden inflammatory process in the pancreas with variable involvement of nearby organs or other organ systems.^[1–3]^ And systemic inflammatory response syndrome is often a complication of severe AP, which leads to high level of inflammatory markers.^[20]^ Patients with severe AP, especially those who require intensive care treatment or mechanical ventilation are prone to develop stress-related acute gastric mucosal lesions.^[21]^ PPIs are the most effective class of drugs used for a variety of acid-related disorders and pantoprazole, as one of PPIs, has been reported that it can reduce tissue infiltration of inflammatory cells and acinar cell necrosis in rats with severe acute pancreatitis.^[10]^ However, the conclusion that PPIs decrease severity or mortality of patients with AP, is controversial.^[15–17]^ In addition, using PPIs may increase the risk for GI infections and the incidence of small intestinal bacteria overgrowth.^[11–14,22]^ To identify the effectiveness and safety of anti-acid therapy with PPIs in AP, we conducted this meta-analysis.

Therefore, there is an urgent requirement to make a systematic review of relevant studies to clarify the effectiveness and safety of anti-acid therapy with PPIs in AP. The results of our review will be reported strictly following the PRISMA criteria. By integrating the data from previous articles, this review will objectively reveal the effectiveness and safety of anti-acid therapy with PPIs in AP.

## Acknowledgments

The authors thank the participants and their families for taking part in the study.

## Author contributions

**Conceptualization:** Tao Cheng, Tian-Yong Han, Haifang Yu, Bofu Liu.

**Data curation:** Tao Cheng, Tian-Yong Han, Bo-Fu Liu, Pan Pan, Zhi-Han Gu.

**Formal analysis:** Tao Cheng, Zhi-Han Gu, Tian-Yong Han, Pan Pan, Bo-Fu Liu.

**Funding acquisition:** Haifang Yu

**Investigation:** Tao Cheng, Bofu Liu, Tian-Yong Han, Zhi-Han Gu, Pan Pan.

**Methodology:** Tao Cheng, Bofu Liu, Tian-Yong Han

**Project administration:** Tao Cheng, Zhi-Han Gu, Pan Pan, Haifang Yu.

**Resources:** Tao Cheng, Zhi-Han Gu, Pan Pan.

**Software:** Tao Cheng, Bo-Fu Liu, Tian-Yong Han.

**Supervision:** Tao Cheng, Zhi-Han Gu, Pan Pan, Haifang Yu.

**Validation:** Tao Cheng, Bo-Fu Liu, Tian-Yong Han, Pan Pan.

**Visualization:** Tao Cheng, Bo-Fu Liu, Zhi-Han Gu.

**Writing – original draft:** Tao Cheng, Bo-Fu Liu, Zhi-Han Gu, Pan Pan, Haifang Yu.

**Writing – review & editing:** Tao Cheng, Haifang Yu.
